# Measuring the resilience of health systems in low- and middle-income countries: a focus on community resilience

**DOI:** 10.1186/s12961-020-00594-w

**Published:** 2020-07-17

**Authors:** Sudip Bhandari, Olakunle Alonge

**Affiliations:** Department of International Health, Johns Hopkins Bloomberg School of Public Health, 615 North Wolfe Street, Baltimore, MD 21205 United States of America

**Keywords:** community resilience, measurement model, vulnerability, health systems

## Abstract

The concept of community resilience has gained considerable attention in the global health discussions since the Ebola outbreak of West Africa in 2014–2015. However, there are no measurement models to quantify community resilience. Without measurement models, it is unclear how to test strategies for building community resilience or to describe their likely intended and unintended results and their impact on health outcomes. We propose a measurement model for community resilience with relevant constructs and indicators to measure these constructs. We conducted a scoping review, systematically searching, screening and selecting relevant articles from two bibliographic databases (PUBMED and Google Scholar) for literature using search terms such as “resilience”, “community resilience” and “health systems resilience”. We screened 500 papers, then completed a full text review of 112 identified as relevant based on their title and abstract. A total of 27 papers and reports were retained for analysis. We then aggregated and synthesised the various definitions of community resilience and the frameworks for understanding these definitions. We identified key constructs from these frameworks and organised these constructs into domains and sub-domains. We proposed indicators to capture aspects of these domains and sub-domains and operationalised these indicators as a measurement model for quantifying community resilience in health systems. We propose a model with 20 indicators to assess community resilience. These indicators tap into various constructs from different theoretical frameworks of community resilience and are useful for assessing the level of knowledge, financial resources, and human, social and physical capital that are needed (or lacking) to respond to any types of shock, including health shock at the community level. This is an initial attempt to describe a multilevel measurement model for quantifying community resilience. This model will help to guide the development and testing of strategies for strengthening community resilience and will require further work to assess its relevance, reliability and validity in different LMIC settings.

## Background

The concept of resilience has gained a lot of attention in global health discussion since the Ebola outbreak in West Africa between 2014 and 2015 [1, 2] due to a recognition of the weakness of national health systems (conceptualised as a lack of resilience) and early failings of global health agencies to adequately respond to the epidemic [3–5]. Kruk et al. [2] identified five characteristics of a resilient health system, including a health system that is aware, diverse, self-regulating, integrated and adaptive, and described these characteristics as an index for monitoring and assessment of national health systems [6]. Other frameworks have similarly been described to understand resilience in health systems [7, 8].

However, the concept of resilience and its related indices in health systems have been criticised for lacking directedness in showing the levels and distributions of health outcomes for a given population [9]. For instance, the concept mostly prioritises maintaining the status quo or stable functioning of health systems and not necessarily beneficial population health outcomes (such as the effective and equitable distribution of health services coverage) [9, 10]. Such criticisms have noted the possibility of maintaining the stability of health systems while perpetuating pre-existing vulnerabilities and societal imbalances that may underlie some currently stable but poorly performing health systems [11, 12]. Furthermore, it is not clear how the concept links to traditional health services and health outcomes, such as universal access to effective health services, equitable distribution of such services and improvement of population health, neither are there measurement models to quantify resilience and its relationships with these outcomes [9]. Without clear measurement models, it is unclear how to develop and test strategies build resilience in health systems or to describe the likely intended and unintended results of these strategies and the impact they may have on health outcomes.

Inasmuch as a health system comprises of actors and activities (or lack thereof) by these actors leading to health outcomes, health system resilience could be conceptualised and measured by some complex aggregation of individual, community and organisation resilience leading to desirable health outcomes [13, 14]. Community resilience particularly has been singled-out as a critical factor for the recovery of health systems in low- and middle-income countries (LMICs) but is often neglected in the assessment of resilience in health systems [15]. For instance, when the Ebola outbreak hit Liberia in 2014–2015, the majority of the external support for addressing the crisis were targeted to an emergency supply of health infrastructure and other structural elements of the health systems. However, studies from Liberia suggested that, while these infrastructural inputs were helpful, the game-changer in stopping the Ebola outbreak were community-led activities and collective actions delivered via existing community structures [4, 15]. The ability to measure community resilience will contribute to efforts to strengthen capacities and structures within communities to prevent or prepare for future health shocks and to achieve positive health outcomes.

In this paper, we propose a measurement model for community resilience in health systems, including relevant constructs and indicators for measuring these constructs. We hope that the model and indicators would allow for quantifying community resilience and facilitate the testing and deployment of effective strategies for strengthening community resilience and equipping communities to reduce the risks of disasters and be better prepared to withstand and address health shocks.

## Methods

### Scoping review

A scoping review of the literature was undertaken between March 2018 and September 2019. Scoping reviews are useful for answering broad research questions, drawing on a comprehensive literature review to identify the nature and extent of research evidence [16]. We performed a scoping review because it allowed us to explore what is known about community resilience and to provide a preliminary assessment of the potential size and scope of the available research literature on this topic, which can serve as the basis for other types of review. The central research question was – what definitions, frameworks and indicators allow for the development of a community resilience measurement model? A five-stage process suggested by Booth et al. [17] was adopted for our search process: (1) initial search of the existing reviews to explore the volume and scope of the literature that is available on the research topic with the goal of identifying databases and key search terms for the search strategy; (2) a systematic search of the peer-reviewed articles as well as grey literature in the databases using the search terms identified in the previous step; (3) a hand-search of the articles by screening the reference lists of all the papers identified in step 2; (4) revision, if necessary, of the research strategy to ensure that all the potentially relevant articles are included to address the research question; and (5) extraction, analysis and recording of relevant information from all the articles to answer the research question.

### Search strategy

We initiated our search by exploring four prior studies on community resilience to develop our search strategy [6, 18–20]. We then used two electronic databases (PubMed and Google Scholar) to systematically search for existing reviews and to comprehensively search for articles as well as reports. For Google Scholar, we combined the following key terms: “community resilience”, “definition”, “indicator”, “framework” and “health system”. For PubMed, we used the MeSH-enriched search terms “resilience” and “community”. We identified 500 peer-reviewed and grey literature (including reports) from the databases search.

### Screening and selection

**Fig. 1:**
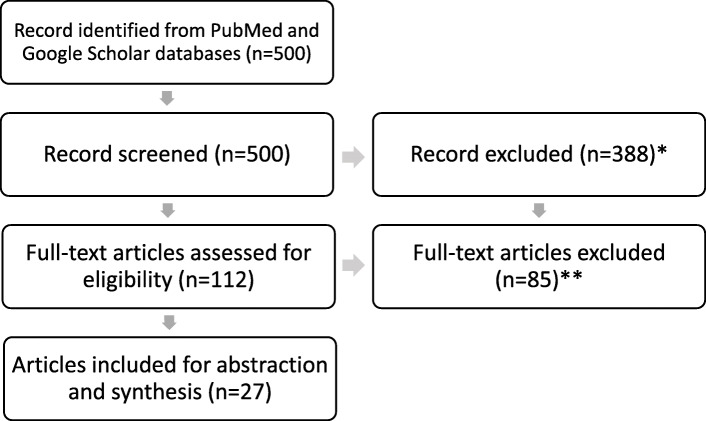
Overview of the review search process flow chart. *Articles excluded for the following reason: title or abstract do not include resilience. **Articles excluded for the following reasons: (1) the title or abstract does not include resilience, community, framework, definitions or variables; (2) the paper is not specific to public health and health systems; (3) the paper does not provide any guidance conceptually or operationally on the definition of community resilience

The first stage of screening and selection consisted of reviewing the title and abstract of the papers identified for the inclusion of the term “resilience”, which resulted in 112 articles meriting further review (Fig. 1). Second, we conducted a full-text review and retained papers if they met the four following criteria: (1) the title or abstract include resilience, community, framework, definitions and variables; (2) the paper is specific to public health and health systems; (3) the paper provides some guidance conceptually or operationally on the topic of community resilience; (4) the paper represents high-income countries (HICs) and/or LMICs. While this review focuses on LMICs, we chose to include papers from HICs in identifying specific attributes of community resilience because some of these attributes (e.g. risk assessment participation and availability of financial resources) apply across both LMIC and HIC settings. We wanted to be comprehensive in our review so as not to miss these broad community resilience attributes. Of the 112 papers, 27 were retained for analysis, 11 of which were related to definitions, 3 to frameworks, and 13 to indicators (see Supplementary Table 1 in the Additional file for a list of these 27 articles retained for abstraction). In order to reduce reviewer bias, screening and selection were first conducted by a single investigator (SB) and the findings were later validated by the second reviewer (OA).

### Data abstraction and synthesis

First, we extracted and summarised data from the 27 included papers on authors, research question, methodology, definitions, frameworks and indicators. Second, the papers were categorised based on their focus on either definition, framework or indicators of the concept of resilience (Table 1). Third, we conducted a thematic analysis of the literature under each of the three focus areas (definition, framework and indicators). We summarised the various definitions of community resilience and compared the key concepts included in those definitions. We prioritised definitions of community resilience that included the most concepts and proposed a definition that incorporates these multiple concepts. We identified theoretical frameworks that have been used to explain the relationships among these various concepts and described the key constructs from these frameworks. We then prioritised constructs that were consistently included by multiple frameworks and organised these constructs into domains and sub-domains. We proposed indicators, identified mainly from the literature, to capture aspects of the constructs included as domains and sub-domains, and operationalised these indicators as a measurement model for quantifying community resilience in health systems. Last, we described how our operationalisation of community resilience could be useful for developing and implementing strategies for strengthening health systems, especially in LMICs.

**Table 1:** Themes included in the thematic analysis

Theme	Description
Definition	Statement that describes the meaning and nature of the concept of resilience
Framework	Theoretical or methodological model that accounts for the relationships among different constructs included within the concept of resilience
Indicators	Items or variables used to empirically measure the constructs

Both the descriptive and thematic analyses were conducted by a single investigator and were validated by the other investigator. The researchers then came together to revise and refine the results of the review.

## Results and discussion

### Definitions of community resilience

With community defined as a geographically bounded entity including people and institutions operating within a common environment, Norris et al. [18] compiled a list of sample definitions of community resilience which we have expanded to include more recent definitions (Table 2). Bond et al. [31] maintain that there are three main commonalities among all the definitions of community resilience. First is the absorption capacity, which aims to identify the magnitude of the shock that a community can withstand and still be able to function in a pre-event scenario. Second is adaptive capacity, which aims to understand the ability of a community to continue to function while adapting to shocks. The third is the restorative capacity, which analyses the ability of a community to get back to its ‘normal’ functioning or pre-event scenario after a shock. Patel et al. [19] found a similar set of commonalities among definitions of community resilience through their systematic literature review. Other authors have also described community resilience as the transformative capacity or the human agency of individuals to limit the impact of shocks and address the vulnerabilities that predispose them to those shocks in the first place [32, 33].

**Table 2:** Representative definitions of community resilience

Citation/Year	Level	Definition
***Process definitions incorporating the absence of adverse effects***		
Sonn, 1998 [21]	Community	The process through which mediating structures (schools, peer groups, family) and activity settings moderate the impact of oppressive systems
Lemyre, 2005 [22]	Individual, Household, Community	A process or the attainment of positive outcomes at the individual, family and community levels despite adversity (e.g. natural disaster, terrorist attack)
Castleden, 2011 [23]	Community	Capability (or process) of a community adapting and functioning in the face of disturbance
***Range of attribute definitions incorporating the absence of adverse effects***		
Brown, 1992 [24]	Community	The ability to recover from or adjust easily to misfortune or sustained life stress
Paton, 2000 [25]	Community	The capability to bounce back and to use physical and economic resources effectively to aid recovery following exposure to hazards
Ganor, 2003 [26]	Community	The ability of individuals and communities to deal with a state of continuous, long-term stress; the ability to find unknown inner strengths and resources in order to cope effectively; the measure of adaptation and flexibility
Ahmed, 2004 [27]	Community	The development of material, physical, socio-political, socio-cultural and psychological resources that promote the safety of residents and buffer adversity
Kimhi, 2004 [28]	Community	Individuals’ sense of the ability of their own community to deal successfully with the ongoing political violence
Coles, 2004 [29]	Community	A community’s capacities, skills and knowledge that allow it to participate fully in recovery from disasters
Pfefferbaum, 2007 [30]	Community	The ability of community members to take meaningful, deliberate collective action to remedy the impact of a problem, including the ability to interpret the environment, intervene and move on
Bond, 2017 [31]	Household, Community	The capacity of a system — a household, a community, an organisation or a coupled natural–human system — to prepare for disruptions from outside of the system, to recover from shocks and stresses, and to adapt and grow from a disruptive experience
***Process definitions combined with a range of attributes and incorporating the absence of adverse effects***		
Norris, 2008 [18]	Community	A process linking a network of adaptive capacities (resources with dynamic attributes) to adaptation after a disturbance or adversity

Community resilience has also been defined by three general types of definitions, which include the ‘process’ definitions that incorporate an ongoing process of change and adaptation, the ‘absence of adverse effect’ definitions, which describe the ability to maintain stable functioning, and the ‘range of attributes’ definition that embraces a community’s broad collection of response-related abilities [19]. For example, Lemyre et al. [22] defined community resilience as “*a process or the attainment of positive outcomes at the individual, family, and community levels despite adversity (e.g., natural disaster, terrorist attack)*”. Similarly, Castleden et al. [23] defined community resilience as a “*capability (or process) of a community adapting and functioning in the face of disturbance*”. Most definitions combine two or more of the general types and can be broadly classified as either process definitions or range of attributes definitions but also incorporating the absence of adverse effects (Table 2).

For this paper, we extend the definition from Norris et al. [18] that combined the three general types of definitions and define community resilience in the health system as a process linking a set of networked adaptive capacities (resources with their dynamic attributes) at individual or community level to a positive trajectory of functioning and adaptation of the health system at the community level after a health shock. This definition bounds the concept of resilience within relevant health delivery and production arrangements at the community level and include adaptive capacities such as interlinked economic resources, social capital, information and communication, and community competence needed for positive health functioning. The extent to which these resources are resistant to depletion (robustness), substitutable (redundancy) and can be readily accessed (rapidity) over time may reflect their dynamic attributes [18]. In addition to combining the general types of definitions (‘process’, ‘absence of adverse effect’ and ‘range of attributes’), this definition acknowledges the dynamic nature and complexities of health systems and resources needed for addressing health shocks with a primary focus on communities. The definition also shows the directedness of health outcomes (i.e. positive trajectory of functioning) and not just resistance or adaptation.

Furthermore, in constraining the definition to the community level, we are able to appropriately operationalise constructs and measures that converge at the same level. We have also adopted a broader definition of health shock in defining community resilience in health systems to include catastrophic events (e.g. epidemics, war and other man-made disasters, and natural disasters such as hurricanes or earthquakes) [34] as well as time-bounded everyday stresses with the potential to disrupt health systems at the community level [35].

### Relevant frameworks for understanding community resilience in health systems

All of the frameworks reviewed for understanding community resilience in health systems overlapped three major frameworks – by Norris et al. [18], Patel et al. [19] and Kruse et al. [20]. The three frameworks capture social constructs and variables employed by other frameworks in conceptualising community resilience both for descriptive and analytical purposes [36–38]. Norris et al.’s [18] framework emphasised the interrelatedness and interdependency among adaptive capacities (including economic development, social capital, information and communication, and community competence) and their dynamic attributes in addressing a shock. Economic development includes the level of economic resources, the degree of equality in the distribution of resources, and the scale of the diversity of those resources within the community. Social capital includes social support (assistance an individual receives from informal networks, e.g. family and friends), social participation (involvement of individuals in formal networks, e.g. professional and religious associations) and community bonds (extent of participation by individuals in community activities, e.g. village festivals, school-organised programmes) [18]. Information and communication include public systems and infrastructure to relay accurate information during and after emergencies and the presence of communal narratives that provide shared meaning and purpose of the information, e.g. telling story of community’s shared experiences and response during and after a crisis. Community competence is similar to human agency but aggregated at a group level and it relates to collective action and decision-making, which can be influenced by the degree of efficacy and level of empowerment of the community to address environmental demands and improve their lives through collaborative efforts [18].

Patel et al. [19] propose breaking down the concept of community resilience into nine different elements that overlapped the adaptive capacities by Norris et al. [18] and adapted in Table 3. However, their framework does not emphasise the system attributes and the interconnectedness of the different elements that may constitute community resilience.

**Table 3:** Elements of community resilience

Elements	Description
Local knowledge	Knowledge that the community possesses about its existing vulnerabilities, which, if addressed prior to a disaster, can improve community resilience This includes: • Factual knowledge base, which relates to knowledge and information acquired in relation to a disaster • Training and education, which is about practices in community education to teach how to respond effectively to an emergency • Collective efficacy and empowerment, which relate to the community’s shared belief in its ability to overcome potential hardships caused by a disaster
Community networks and relationships	The connectedness and cohesiveness of community members during a crisis. Connectedness, also called ‘social network’ can be examined through linkages within a community. Cohesiveness can be based on these linkages and are described as weak or strong ties. Factors like trust and shared values can improve ties and consequently community resilience
Communication	Communication includes: • Effective communication: this means that the community has opportunities for open dialogue and has established infrastructure that could be coordinated in a pre- or post-disaster setting • Risk communication: this deals with the provision of accurate and culturally acceptable information about possible threats • Crisis communication: this includes the provision of up-to-date information about the ongoing impact and relief efforts in real-time using traditional and social media
Health	Health encompasses the pre-existing health of a community and the delivery of health services after a disaster. Health services include short-term and long-term delivery of quality physical and mental health services, which can be improved through training and capacity-building at the hospital and facility level to handle mass casualties
Governance	Governance focus on how communities coordinate and handle emergencies. This includes: • Infrastructure and services: this relates to whether the community has effective, efficient and capable infrastructure and services to handle crises; for example, infrastructure should be able to handle incoming information about an emergency and send instructions and implement a response during and after a disaster • Public involvement and support: a community’s involvement in strategic planning, response and recovery as they relate to the uniqueness and aspirations of the community
Resources	Resources include tangible supplies (food, water, first aid kits), technical resources (shelter, automobiles, machinery) and even financial as well as social resources

Kruse et al. [20] described three intertwined domains that form the core of community resilience. The domains include resources and capacities, actions, and learning domains. The resources and capacities domain very much overlap the adaptive capacities described by Norris et al. [18] and the nine elements by Patel et al. [19]. The actions domain comprises of resilient actions that communities may undertake to prevent or limit the impact of natural hazards, including hazard-specific activities (e.g. civil protection actions such as weather forecasting) and hazard-independent activities (e.g. social protection actions such as the provision of social protection amenities like food banks, shelters, and community or emergency funds accessible to vulnerable populations). The learning domain focuses on ongoing formal and informal learning embedded within social networks that enables the community to detect and collectively respond to hazards. Such learning includes awareness of potential, current or past hazards, ability to recognise when hazards require immediate response, opportunities for testing different innovative approaches for addressing the hazards, disseminating of effective approaches, and monitoring and review of existing processes for mitigating hazards within the community. The domains by Kruse et al. [20] are intertwined to emphasise that they are intrinsically linked to contribute to community resilience. The Kruse et al. [20] framework further describes context-specific factors for natural disaster management (that may be external to the community), which may not be readily transportable for discussing other types of shocks.

### A proposed measurement model for quantifying community resilience in health systems

To generate a measurement model that will operationalise the constructs included in our definition of community resilience, we combine the three major frameworks discussed above by including all of the constructs that they described in a single framework (Tables 4, 5, 6 and 7). Various domains and sub-domains within Norris et al.’s [18] framework can be connected to the constructs that Patel et al. [19] described in their resilience framework, and these are mainly capacities which also overlapped with resources and capacities domain covered by the Kruse et al. [20] framework. We then propose indicators for these constructs based on the review and reflect on the dynamic attributes of these indicators, that is, their robustness, redundancy and rapidity over time (Tables 4, 5, 6 and 7). While reflecting on the dynamic attributes of the indicators, we make the assumption that these indicators are being used in low-income and disaster settings. We recognise that there are obvious limitations of the assumptions; for example, these descriptions may not apply in high-income or have a limited application in settings of routine, multiple challenges (which require everyday resilience [20]). Further, we incorporated constructs from two domains (action and learning) in the Kruse et al. [20] framework to explicitly describe constructs related to actions to mitigate health shocks and activities that facilitate ongoing learning that enhances collective response to health shocks in communities (Tables 4, 5, 6 and 7).

**Table 4:** Juxtaposition of the ‘economic development’ domain and sub-domains of Norris et al.’s [18] framework with Patel et al.’s [19] and Kruse et al.’s [20] elements for community resilience

**Norris framework**		**Patel framework**		**Kruse framework**		**Example of primary indicators**	**Dynamic attributes of indicators (robust, redundant, rapid)** **• Robust:** resistant to depletion **• Redundant:** substitutable **• Rapid:** can be readily accessed	**Positive health trajectory?** Whether the indicator shows positive influences on health outcomes and the equity of those outcomes
**Domains**	**Sub-domains**	**Elements that relate to the Norris’ domain and sub-domains**	**Description of elements that relate to the domain and/or sub-domain**	**Elements that relate to Norris’s domain and sub-domains**	**Description of elements that relate to the domain and/or sub-domain**			
Economic Development	Resource Volume	Resources	Include tangible supplies, financial and technical resources as well as social resources that support livelihood and relevant for mitigating shocks within a community	Domain: Resources and capacities Element: Natural/place-based capacities, financial, physical capacities, human capacities	Include availability of natural resources, e.g. land, water, forest, as well as local public services, amenities and access to markets; financial resources, e.g. money and credit facilities; physical capacities, e.g. adequate roads, water, housing and sanitation; individual capacities, including health status, education and skills to mitigate shocks	**Physical resource:** Percentage of households with year-round access to clean water [37]	**Physical resource:** not robust, redundant or rapid Physical resources can deplete easily in disaster situations (not robust), especially when demand for such resources might be higher and supply might be hindered due to demolition; some of these resources, like clean water or food, might not be substitutable either simply because they are necessary for survival (not redundant); lastly, rapid access to these resources would be challenging in a crisis situation where supply might be dislodged (not rapid)	No
						**Human resource:** number of household members with secondary education or higher [37]	**Human resource:** not robust or redundant but rapid; human resources may be limited due to disability and death due to disasters (not robust); skilled human resources required to address a crisis situation may also not be substitutable (not redundant); however, it might be possible to readily access the human resource in a community (rapid)	No
						**Financial resource:** disaster relief fund per capita [38]	**Financial resource:** not robust or rapid but redundant Financial resources, similar to physical resources, can be readily depleted in crisis situations (not robust); however, there might be multiple organisations that provide relief funds to address the demand for food, shelter, water and other needs; therefore, these resources could be substitutable (redundant); these resources, though available, may not be readily accessible to the community due to legal barriers, destruction of financial infrastructure due to disasters, or unavailability of human resources to process financial transactions (not rapid)	No
	Resource diversity	Resources	Extent to which resources are not limited to a narrow range of options within a community	Not applicable	Not applicable	**Emergency services information:** Presence and type of emergency service [39]	**Emergency services information:** robust, redundant and rapid Information on emergency services and preparedness developed to address disasters could be available in magazines, reports and online; distribution and access to these resources for the community requires minimal cost (robust); these resources are easily substitutable (redundant); lastly, they can be accessed without delay in crisis situations as individual families could have a copy of the resource or might be able to retrieve it through online systems (rapid)	No
	Resource Equity and Social Vulnerability	Resources	Entails distributive justice – ensuring the fairness of resource allocation and the ability of the community to harness resources	Not applicable	Not applicable	**Local planning involvement:** Percentage of households with women and marginalised groups involved in local planning processes [37]	Local planning involvement: not robust or redundant but rapid Households with women and marginalised groups involved in local planning processes might be limited due to disability and death due to disasters (not robust); these women and marginalised groups might be crucial in getting other women and marginalised populations on board to respond to crises; therefore, they are not substitutable either (not redundant); however, they might be rapid as it is possible to readily access these households as they are situated in the community (rapid)	Yes
						**Risk assessment participation:** Level of participation of vulnerable groups in the risk assessment [40]	**Risk assessment participation:** not robust or redundant but rapid Level of participation might dwindle in crisis situations due to disability and deaths during disasters; tending to immediate personal and family needs might be prioritised over community needs (not robust); participation of these groups is not substitutable given their importance in getting other vulnerable community members on board (not redundant); however, participation can be readily accessed as these groups are present in the community (rapid)	Yes

**Table 5:** Juxtaposition of the ‘social capital’ domain and sub-domains of Norris et al.’s [18] framework with Patel et al.’s [19] and Kruse et al.’s [20] elements for community resilience

**Norris framework**		**Patel framework**		**Kruse framework**		**Example of primary indicators**	**Dynamic attributes of indicators (Robust, Redundant, Rapid)** **• Robust:** resistant to depletion **• Redundant:** substitutable **• Rapid:** can be readily accessed	**Positive health trajectory?** Whether indicator shows positive influences on health outcomes and the equity of those outcomes
**Domains**	**Sub-domains**	**Elements that relate to the Norris’ domain and sub-domains**	**Description of elements that relate to the domain and/or sub-domain**	**Elements that relate to Norris’s domain and sub-domains**	**Description of elements that relate to the domain and/or sub-domain**			
Social Capital	Network Structures and Linkages	Community networks and relationships	Encompasses the connectedness of community members during a crisis Connectedness, also called ‘social network’ can be examined through linkages within a community	Not applicable	Not applicable	**National platform participation:** Participation by type and objective of NGOs, civil society, volunteers and the private sector in community platforms [41]	**National platform participation:** not robust or redundant but rapid Level of participation by various groups might dwindle in crisis situations due to disability and deaths during disasters; therefore, it is not resistant to depletion (not robust); participation of these groups is not substitutable either as their coalition would be important to tap into different strengths and resources to respond to crisis situations (not redundant); participation can be readily accessed as these groups are present in the community (rapid)	Yes
						**Civic organisations:** Number of civic organisations per 10,000 population [42]	Civic organisations: not robust or redundant but rapid While the number of civic organisations that are registered might not dwindle, those in leadership and management may deplete due to disability and deaths, which would make the organisations defunct (not robust); these civic organisations are not substitutable either as they might be important to deliver resources and provide relief during crises situation (not redundant); however, participation can be readily accessed either as these organisations are present in the community (rapid)	No
	Community bond	Community networks and relationships	Includes cohesiveness, which can be based on community linkages and are described as weak or strong ties; factors like trust and shared values can improve ties	Not applicable	Not application	**Community participation:** Proportion of individuals who actively engage in community-based associations or events, e.g. farmers’ association and new harvest celebration in the agrarian community [33]	**Community participation:** not robust, redundant or rapid Community participation may wax and wane depending on living conditions and sentiments of individuals that are members of that community (not robust) and such participation is not substitutable by other kinds of activities (not redundant); it takes time to develop the relationships that lead to such participation or access their benefits in a time of a crisis (not rapid)	No
	Social Support	Social support	Includes assistance such as food and monetary that individuals are able to draw upon from informal networks, e.g. family and friends	Domain: Action Element: Social protection	Includes various actions that provide community members with the resources necessary to improve their living standards; success of social protection mechanisms is dependent on the strength of social support systems, including the presence of an active community-based voluntary sector capable of providing social support at times of disaster	**Family support:** proportion of individuals willing to provide food and monetary support to non-close family members [43]	**Family support:** not robust but redundant and rapid Family support may be depleted, especially if those family members are also facing adverse situations (not robust), but can be substituted or augmented with support from formal networks, e.g. relief and humanitarian organisations (redundant) and can be rapidly accessed in time of a crisis (rapid)	No
						**Voluntary workers:** Percentage of voluntary workers for an organisation or group (persons over 15) [44]	**Voluntary workers:** not robust or redundant but rapid Similar to other human resources, voluntary workers for an organisation or group might dwindle during crisis situations due to death and disability (not robust); volunteers are not substitutable given their importance in relief efforts during crisis and rebuilding afterward (not redundant); similar to other human resources, volunteers (those who are not directly affected by the crisis) could be readily accessed since they are present in the community (rapid)	No
	Community Bonds, Roots and Commitments	Community networks and relationships	Includes community linkages that are determined by trust and shared values; these conceptually focus on bonding, bridging and linking	Domain: Resources and capacities Element: Socio-political	Relates to the importance of political, social and power dynamics of community members; refers to lateral relationships between family, friends and informal networks as well as formal membership groups with institutional hierarchies	**Vulnerable people inclusion:** Number of vulnerable (e.g. marginalised) people included in formal and informal networks [45]	**Vulnerable people inclusion:** not robust or redundant but rapid See the explanation above for “percentage of households with women and marginalised groups involved in local planning processes [37]” indicator above	Yes

**Table 6:** Juxtaposition of the ‘information and communication’ domain and sub-domains of Norris et al.’s [18] framework with Patel et al.’s [19] and Kruse et al.’s [20] elements for community resilience

**Norris framework**		**Patel framework**		**Kruse framework**		**Example of primary indicators**	**Dynamic attributes of indicators (Robust, Redundant, Rapid)** • **Robust:** resistant to depletion • **Redundant:** substitutable • **Rapid:** can be readily accessed	**Positive health trajectory?** Whether indicator shows positive influences on health outcomes and the equity of those outcomes
**Domains**	**Sub-domains**	**Elements that relate to the Norris’ domain and sub-domains**	**Description of elements that relate to the domain and/or sub-domain**	**Elements that relate to Norris’s domain and sub-domains**	**Description of elements that relate to the domain and/or sub-domain**			
Information and Communication	Systems and Infrastructure for Informing the Public	Communication	Involves established infrastructure that could be coordinated in a pre- or post-disaster setting; includes crisis communication, which entails the provision of up-to-date information about the ongoing impact and relief efforts in real-time using traditional and social media	Not applicable	Not applicable	**Early warning systems:** Number of community with early warning systems in place [45]	**Early warning systems:** not robust or redundant but rapid This indicator may not be robust given that communities with early warning systems in place could be affected by crises, depending on the magnitude of the crisis (not robust); these may not be redundant either given the importance of having these early warning systems to build the resilience of communities (not redundant); however, communities with these early warning systems could be readily accessed if they are not severely affected by the crises (rapid)	No
						**Communication means:** Robust and extended communication means available throughout areas at risk [41]	**Communication means:** not redundant but robust and rapid Communication methods can vary and could be resistant to depletion. For example, information available publicly online could be accessed and does not reduce on higher consumption (robust); these communication means may not be substitutable as these means could be set up to align with the community needs and infrastructure availability (not redundant); if these communication means are not severely hampered, these can be readily accessed and provide valuable information about the impact of crises and relief efforts (rapid)	Yes
	Communication and Narrative	Communication	Includes risk communication, which deals with the provision of accurate and culturally acceptable information about possible threats			**Population alerts:** Early warning information and alerts reaching populations at risk [41]	**Population alerts:** not redundant but robust and rapid See explanations for the indicator “Robust and extended communication means available throughout areas at risk”	Yes

**Table 7:** Juxtaposition of the ‘community competence’ domain and sub-domains of Norris et al.’s [18] framework with Patel et al.’s [19] and Kruse et al.’s [20] elements for community resilience

**Norris framework**	**Patel framework**			**Kruse framework**		**Example of primary indicators**	**Dynamic attributes of indicators (Robust, Redundant, Rapid)** **• Robust:** resistant to depletion **• Redundant:** substitutable **• Rapid:** can be readily accessed	**Positive health trajectory?** Whether indicator shows positive influences on health outcomes and the equity of those outcomes
**Domains**	**Sub-domains**	**Elements that relate to the Norris’ domain and sub-domains**	**Description of elements that relate to the domain and/or sub-domain**	**Elements that relate to Norris’s domain and sub-domains**	**Description of elements that relate to the domain and/or sub-domain**			
Community Competence	Collective Action and Decision-making	Governance and Leadership	Includes public involvement and support, which is about the community’s involvement in strategic planning, response and recovery as they relate to the uniqueness and aspirations of the community	Domain: Learning Element: Critical reflection	Success of social learning tends to be dependent on how embedded a practice is in social networks; critical reflection allows space for social interaction among community members and deliberation on the risk-related social contract of the community, which leads to better decision-making and collective action	**Self-efficacy** Percentage of individuals who perceive more control or influence in their community based on prior learning experience [46]	**Self-efficacy:** neither robust or redundant but rapid Community competence derives from the collection of competent individuals who can navigate at times of health shocks; they might be affected due to disability and death due to disasters (not robust); depending on their availability, they may not be substitutable either, as these individuals might be crucial in directing other populations to respond to crises (not redundant); however, they might be readily accessed given their availability within the community (rapid)	Yes
				Domain: Action Element: Civil protection	Focuses on actions taken by the community on phases of the disaster management cycle (preparedness, response, recovery and mitigation)	**Leadership:** Land-use plans that have been developed with reference to local hazard risk assessment and that have been subjected to a formal consultation processes [47]	**Leadership:** redundant, robust and rapid (See the explanation for “presence and type of emergency service community engagement strategy” above)	No
						**Simulation exercises:** Number of simulation exercises conducted [36]	**Simulation exercises:** not redundant but robust and rapid Unlike physical or financial resources, simulation exercises that have been conducted are resistant to depletion because communities might incorporate these exercises into their behaviours and crisis plans (robust); however, these exercises are not redundant as they might be unique to particular communities’ needs and resource availability (not redundant); these exercises can be readily accessed because individuals involved in these exercises would be present in the community at times of crisis (rapid)	No
						**Contingency plans:** Number of community contingency plans in place [45]	**Contingency plans:** not redundant but robust and rapid See the explanation for the indicator “Robust and extended communication means available throughout areas at risk”	Yes
	Collective Efficacy and Empowerment	Local Knowledge	Includes collective efficacy and empowerment, which is about the community’s shared belief of its ability to overcome potential hardships through self-reliance	Domain: Action Element: Social protection	Includes consideration of how the provision of welfare services (e.g. education, housing, health, etc.) improves the community’s capacity to reduce the livelihood risks faced by some in the community	**Responsible agencies**: Location and level by type of responsible designated agencies, institutions and offices for the implementation of enforcement system [41]	**Responsible agencies:** not redundant or robust but rapid (See the explanation for the indicator “Number of civic organisations per 10,000 population” above)	1. No

### How our operationalisation of community resilience could be useful for developing and implementing strategies for strengthening health systems

We propose 20 indicators to assess community resilience (Tables 4, 5, 6 and 7) and these indicators tap into various constructs from different theoretical frameworks previously used to describe the concepts [30, 36–47]. These indicators are selected from various reports and peer-reviewed papers that address aspects of resilience from different sectors. For example, the ‘presence and type of emergency services’ indicator is based on a report by researchers in the emergency management community in Australia. They found the need and opportunity to operationalise ideas of disaster resilience after the Australian government published the ‘National Strategy for Disaster Resilience’.

While each indicator in our table was operationalised for specific subject areas and the context in which these documents were developed, we deemed them to be versatile enough to be used in other settings. For example, in the case of ‘presence and type of emergency services’ indicator described above, this measure was conceptualised to understand the availability of natural hazard information, community engagement and partnerships to encourage risk awareness in Australia. This measure is adaptable enough to be used in other subject areas and contexts to measure approaches, including information, participation, consultation and empowerment of communities — critical pieces of community resilience.

These indicators are useful for assessing the level of knowledge, financial resources, and human, social and physical capital that are needed (or lacking) to respond to any types of shock, including health shock at the community level. Some of these indicators are assessed at the household level, e.g. percentage of households with access to physical resources, while others are assessed at the community level, e.g. presence of early warning systems and robust communication channels, and they can provide a useful snapshot for developing strategies for strengthening community resilience in LMICs.

These indicators are regarded as primary indicators because they could be further operationalised to elicit the dynamic attributes of their constructs. For example, the percentages of household with year-round access to clean water elicit information about the physical resources needed for survival and could be further operationalised to consider the percentage of households with access over multiple years (robustness), whether there are multiple sources for clean water or not (redundancy) or whether clean water is delivered directly to the point of use or the round-trip time for fetching water is less than 30 minutes [48] (rapidity).

The ability to elicit specific measures for different constructs of community resilience using these indicators would allow for comparison of the level of community resilience among populations and provide an opportunity for understanding how certain communities are more resilient than others and test specific hypotheses around factors that contribute to these differences. Each of the indicators provides a direct focal point for strategies for improving a specific domain of community resilience, e.g. the lack of a robust communication channel at the community level may necessitate the strengthening of existing or development of new communication platforms, e.g. local radio, town criers and other traditional announcement platforms, to reach vulnerable populations in hard-to-reach places.

The indicators would also help to clarify the pathways for how strategies for strengthening community resilience contribute to better health outcomes since the success or failures of these strategies can be assessed by the indicator, which could be clearly mapped to health indicators. For example, strategies to build the capacity of town criers and messengers to relay accurate and timely health information about a risk factor can be readily linked to the health indicator assessing the prevalence of the risk factor or the health problem that may occur as a consequence of that risk factor.

Community resilience can be measured focusing on one or two relevant indicators (out of the list of 20 indicators) and further operationalised to demonstrate their dynamic attributes among a given population. The interactions among the dynamic attributes for each indicator can also be explored to create complex indices that show the system features of the related constructs. Furthermore, comprehensive measures that combine most or all of the indicators can be determined and the interactions among multiple relevant indicators could also be further explored for a given health shock. For example, an assessment of all the 20 indicators could be done for a given population and combined into comprehensive or complex measures that would allow for comprehensive analysis of gaps in community resilience, assessing the level of preparedness of communities to shocks, and the development of multi-faceted strategies that address domains of community resilience at multiple socioecological levels.

While we have attempted to capture the different domains of community resilience using the proposed indicators, the indicators may be inadequate to capture all of the different aspects that contribute to the specific construct that those indicators are supposed to measure and may also not be able to show contextually determined variations in the level of these different aspects. For example, the indicator on the number of individuals from vulnerable groups included in a specific formal or informal network may not be sufficient to distinguish those who benefited from either network, which may be an important distinction to make in assessing community resilience in a given context. Some of the indicators may also not be readily measurable and data may be lacking for capturing some of these indicators. For the next steps, these indicators will be assessed for different country contexts and types of health shocks. Such assessment would provide guidance for revising the indicators, or developing new indicators, and determining the relevance of the indicators for assessing community resilience in different contexts. It would also provide an opportunity for assessing the validity and reliability of the indicators.

Our study has a few limitations. First, it is based on a scoping review design, which does not entail assessing the quality of the included papers. Unlike some other types of reviews (e.g. systematic reviews), a scoping review does not involve formal quality assessments. Additionally, due to the lack of consensus on methodological standards for scoping review designs, scholars may disagree with some of the steps we have undertaken as part of our review process. We made an attempt to address the issue regarding methodological standards by adopting a scoping review process described by one of the most frequently cited guidelines for conducting scoping reviews [16]. The second limitation relates to our inclusion/exclusion criteria. We limited our papers to include those that relate to public health and health systems and this may have potentially led to the exclusion of papers from other disciplines (e.g. geography) [49]. However, the domains and sub-domains described in resilience frameworks from these other disciplines significantly overlap the domains reviewed in our included papers that focus on health since most of these domains are multisectoral and are relevant for understanding complex factors that underlie community resilience in health systems.

## Conclusion

This study presents a synthesis of definitions of community resilience and frameworks for understanding resilience, and proposes a set of indicators that can be used for assessing community resilience. These indicators are useful for guiding the development of strategies for strengthening communities, assessing the readiness and preparedness of communities to respond to health shocks, prioritising resources for addressing shocks, and linking resilience investment and outcomes to traditional health systems outcomes such as equity and effective health services in LMICs. The indicators are a first attempt to describe a multilevel measurement model for quantifying community resilience and will require further work to assess their relevance, reliability and validity in different LMIC settings.

## Supplementary information

**Additional file 1: Supplementary Table 1.** List of articles retained for abstraction against the selection criteria. Supplementary table to support the conclusions of this article.

## Data Availability

Not applicable.

## Abbreviations

HICs: high-income countries
LMICs: low- and middle-income countries
