# Supplementary material for: Measuring the resilience of health systems in low- and middle-income countries: a focus on community resilience
Source: Health Res Policy Syst. 2020 Jul 17;18:81. doi: 10.1186/s12961-020-00594-w (PMC7368738; doi:10.1186/s12961-020-00594-w)
Supplement: Supplementary file 1 — Additional file 1: Supplementary Table 1. List of articles retained for abstraction against the selection criteria. Supplementary table to support the conclusions of this article. [file 12961_2020_594_MOESM1_ESM.docx]

**Supplementary Table 1:** List of articles retained for abstraction against the selection criteria

| **S.N.** | **Author & Year** | **Title** | **The title or abstract include resilience, community, framework, definitions, variables** | **The paper is specific to public health and health systems** | **The paper provides some guidance conceptually or operationally on the topic of community resilience** | **The paper represents low- and middle- income countries** |
| --- | --- | --- | --- | --- | --- | --- |
|  |  |  | **Yes/No** | **Yes/No** | **Yes/No** | **Yes/No** |
| 1. | (Norris et al., 2008) | Community Resilience as a Metaphor, Theory, Set of Capacities, and Strategy for Disaster Readiness | Y | Y | Y | Y |
| 2. | (Patel et al., 2017) | What Do We Mean by ‘Community Resilience’? A Systematic Literature Review of How It Is Defined in the Literature | Y | Y | Y | Y |
| 3. | (Kruse et al., 2017) | Conceptualizing community resilience to natural hazards – the emBRACE framework | Y | Y | Y | Y |
| 4. | (Bond et al., 2017) | Resilience Dividend Valuation Model Framework Development and Initial Case Studies | Y | Y | Y | Y |
| 5. | (B. Pfefferbaum et al., 2015) | Community Resilience Interventions: Participatory, Assessment-Based, Action-Oriented Processes | Y | Y | Y | N |
| 6. | (Lemyre et al., 2005) | A Psychosocial Risk Assessment and Management Framework to Enhance Response to CBRN Terrorism Threats and Attacks | Y | Y | Y | N |
| 7. | (Castleden et al., 2011) | Resilience thinking in health protection | Y | Y | Y | N |
| 8. | (Sonn & Fisher, 1998) | Sense of community: Community resilient responses to oppression and change | Y | Y | Y | Y |
| 9. | (Brown & Perkins, 1992) | Disruptions in Place Attachment | Y | Y | Y | Y |
| 10. | (Paton et al., 2001) | Community Resilience to Volcanic Hazard Consequences | Y | Y | Y | N |
| 11. | (Ganor & Ben-Lavy, 2003) | Community Resilience: Lessons Derived from Gilo Under Fire | Y | Y | Y | N |
| 12. | (Ahmed et al., 2004) | Discerning Community Resilience in Disadvantaged Communities in the Context of Violence and Injury Prevention | Y | Y | Y | Y |
| 13. | (Kimhi & Shamai, 2004) | Community resilience and the impact of stress: Adult response to Israel’s withdrawal from Lebanon | Y | Y | Y | Y |
| 14. | (Coles & Buckle, 2004) | Developing community resilience as a foundation for effective disaster recovery | Y | Y | Y | N |
| 15. | (B. J. Pfefferbaum et al., 2007) | Building Resilience to Mass Trauma Events | Y | Y | Y | N |
| 16. | (Rose, 2004) | Defining and measuring economic resilience to disasters | Y | Y | Y | N |
| 17. | (Francis & Bekera, 2013) | A metric and frameworks for resilience analysis of engineered and infrastructure systems | Y | Y | Y | N |
| 18. | (Bruneau et al., 2003) | A Framework to Quantitatively Assess and Enhance the Seismic Resilience of Communities | Y | Y | Y | N |
| 19. | (UNDP, 2017) | Community Based Resilience Analysis (CoBRA) Conceptual Framework and Methodology | Y | Y | Y | Y |
| 20. | (Yoon et al., 2016) | A measurement of community disaster resilience in Korea | Y | Y | Y | N |
| 21. | (Morley et al., 2015) | The Australian Natural Disaster Resilience Index | Y | Y | Y | N |
| 22. | (W.A. & Kafle, 2009) | Integrated Community Based Risk Reduction: An Approach to Building Disaster Resilient Communities | Y | Y | Y | Y |
| 23. | (United Nations, 2008) | Guidance on Measuring the Reduction of Disaster Risks and the Implementation of the Hyogo Framework for Action | Y | Y | Y | Y |
| 24. | (Morrow, 2008) | Community resilience: A social justice perspective | Y | Y | Y | N |
| 25. | (The Rockefeller Foundation & ARUP, 2014) | City Resilience Framework | Y | Y | Y | Y |
| 26. | (Perfrement & Lloyd, 2015) | The Composite Resilience Index—A modelling tool to measure the resilience of local communities to climate extremes | Y | Y | Y | N |
| 27. | (Torgerson, 2017) | Measuring Community Action Program Impacts on Multi-Dimensional Poverty | Y | Y | Y | N |

**REFERENCES**

Ahmed, R., Seedat, M., van Niekerk, A., & Bulbulia, S. (2004). Discerning Community Resilience in Disadvantaged Communities in the Context of Violence and Injury Prevention. *South African Journal of Psychology*, *34*(3), 386–408. https://doi.org/10.1177/008124630403400304

Bond, C. A., Strong, A., Burger, N., Weilant, S., Saya, U., & Chandra, A. (2017). *Resilience Dividend Valuation Model Framework Development and Initial Case Studies*.

Brown, B. B., & Perkins, D. D. (1992). Disruptions in Place Attachment. In I. Altman & S. M. Low (Eds.), *Place Attachment* (pp. 279–304). Springer US. https://doi.org/10.1007/978-1-4684-8753-4_13

Bruneau, M., Chang, S. E., Eguchi, R. T., Lee, G. C., O ’rourke, T. D., Reinhorn, A. M., Shinozuka, M., Tierney, K., Wallace, W. A., & Von Winterfeldt, D. (2003). *A Framework to Quantitatively Assess and Enhance the Seismic Resilience of Communities*. https://doi.org/10.1193/1.1623497

Castleden, M., McKee, M., Murray, V., & Leonardi, G. (2011). Resilience thinking in health protection. *Journal of Public Health*, *33*(3), 369–377. https://doi.org/10.1093/pubmed/fdr027

Coles, E., & Buckle, P. (2004). *Developing community resilience as a foundation for effective disaster recovery*.

Francis, R., & Bekera, B. (2013). A metric and frameworks for resilience analysis of engineered and infrastructure systems. *Reliability Engineering and System Safety*, *121*, 90–103. https://doi.org/10.1016/j.ress.2013.07.004

Ganor, D. M., & Ben-Lavy, Y. (2003). *Community Resilience: Lessons Derived from Gilo Under Fire*.

Kimhi, S., & Shamai, M. (2004). Community resilience and the impact of stress: Adult response to Israel’s withdrawal from Lebanon. *Journal of Community Psychology*, *32*(4), 439–451. https://doi.org/10.1002/jcop.20012

Kruse, S., Abeling, T., Deeming, H., Fordham, M., Forrester, J., Jülich, S., Karanci, A. N., Kuhlicke, C., Pelling, M., Pedoth, L., & Schneiderbauer, S. (2017). Conceptualizing community resilience to natural hazards – the emBRACE framework. *Natural Hazards and Earth System Sciences*, *17*(12), 2321–2333. https://doi.org/10.5194/nhess-17-2321-2017

Lemyre, L., Clément, M., Corneil, W., Craig, L., Boutette, P., Tyshenko, M., Karyakina, N., Clarke, R., & Krewski, D. (2005). A Psychosocial Risk Assessment and Management Framework to Enhance Response to CBRN Terrorism Threats and Attacks. *Biosecruity and Bioterroristm: Biodefense Strategy, Practice, and Science*, *3*(4).

Morley, P., Parsons, M., Marshall, G., Hastings, P., Glavac, S., Stayner, R., McNeill, J., & Reeve, I. (2015). *The Australian Natural Disaster Resilience Index*. https://www.bnhcrc.com.au/publications/biblio/bnh-2345

Morrow, B. H. (2008). *Community resilience: A social justice perspective*. http://rgdoi.net/10.13140/RG.2.1.1278.9604

Norris, F. H., Stevens, S. P., Pfefferbaum, B., Wyche, K. F., & Pfefferbaum, R. L. (2008). Community Resilience as a Metaphor, Theory, Set of Capacities, and Strategy for Disaster Readiness. *American Journal of Community Psychology*, *41*(1–2), 127–150. https://doi.org/10.1007/s10464-007-9156-6

Patel, S. S., Rogers, M. B., Amlôt, R., & Rubin, G. J. (2017). What Do We Mean by ‘Community Resilience’? A Systematic Literature Review of How It Is Defined in the Literature. *PLOS Currents Disasters*. https://doi.org/10.1371/currents.dis.db775aff25efc5ac4f0660ad9c9f7db2

Paton, D., Millar, M., & Johnston, D. (2001). *Community Resilience to Volcanic Hazard Consequences*. *24*, 157–169.

Perfrement, T., & Lloyd, T. (2015). *The Composite Resilience Index—A modelling tool to measure the resilience of local communities to climate extremes*. https://theresilienceindex.weebly.com/our-solution.html

Pfefferbaum, B. J., Reissman, D. B., Pfefferbaum, R. L., Klomp, R. W., & Gurwitch, R. H. (2007). Building Resilience to Mass Trauma Events. In L. S. Doll, S. E. Bonzo, D. A. Sleet, & J. A. Mercy (Eds.), *Handbook of Injury and Violence Prevention* (pp. 347–358). Springer US.

Pfefferbaum, B., Pfefferbaum, R. L., & Van Horn, R. L. (2015). Community Resilience Interventions: Participatory, Assessment-Based, Action-Oriented Processes. *American Behavioral Scientist*, *59*(2), 238–253. https://doi.org/10.1177/0002764214550298

Rose, A. (2004). Defining and measuring economic resilience to disasters. *Disaster Prevention and Management: An International Journal*. https://doi.org/10.1108/09653560410556528

Sonn, C. C., & Fisher, A. T. (1998). *Sense of community: Community resilient responses to oppression and change*. 15.

The Rockefeller Foundation, & ARUP. (2014). *City Resilience Framework*. https://assets.rockefellerfoundation.org/app/uploads/20140410162455/City-Resilience-Framework-2015.pdf

Torgerson, M. (2017). *Measuring Community Action Program Impacts on Multi-Dimensional Poverty*. Oregon State University. https://ruralstudies.oregonstate.edu/sites/agscid7/files/rsp_1701.pdf

UNDP. (2017). *Community Based Resilience Analysis (CoBRA) Conceptual Framework and Methodology*. https://www.undp.org/content/undp/en/home/librarypage/environment-energy/sustainable_land_management/CoBRA/cobra_guide.html

United Nations. (2008). *Guidance on Measuring the Reduction of Disaster Risks and the Implementation of the Hyogo Framework for Action*. https://www.unisdr.org/files/13101_ImplementingtheHFA.pdf

W.A., F., & Kafle, S. K. (2009). *Integrated Community Based Risk Reduction: An Approach to Building Disaster Resilient Communities* (4th Annual International Workshop & Expo on Sumatra Tsunami Disaster & Recovery).

Yoon, D. K., Kang, J. E., & Brody, S. D. (2016). A measurement of community disaster resilience in Korea. *Journal of Environmental Planning and Management*, *59*(3), 436–460. https://doi.org/10.1080/09640568.2015.1016142
